# A Scoping Review on Quality Physical Education Programmes and Their Outcomes on Primary-Level Pupils

**DOI:** 10.3390/ijerph20043575

**Published:** 2023-02-17

**Authors:** Úna Kingston, Manolis Adamakis, Diarmuid Lester, João Costa

**Affiliations:** 1 Sports Studies and Physical Education Programme, School of Education, University College Cork, T12 CY82 Cork, Ireland; 2 School of Physical Education and Sport Science, National and Kapodistrian University of Athens, 10556 Athens, Greece

**Keywords:** quality physical education, programmes, primary education, academic achievement, attitudes, wellbeing, physical activity, fidelity

## Abstract

A scoping review was carried out on the literature relating to the evaluation of the implementation of quality physical education (QPE) programmes and related outcomes on final-stage primary-level pupils’ attitudes towards physical education (ATPE), physical activity behaviour (PAB), mental wellbeing (MWB) and academic achievement (AA). The scoping review included studies published between 2000 and 2020 in the PubMed, Elsevier, SCOPUS and CINAHL databases and was completed in accordance with the PRISMA extension for scoping reviews’ guidelines. Based on the inclusion criteria, 15 out of 2869 studies were included in the review. A thematic analysis was used to inductively and deductively analyse the studies for common themes of features of QPE programmes in primary schools, arising from nine different countries, considering the four outcome dimensions (ATPE, PAB, MWB and AA). The common themes identified as features of QPE across all four dimensions were as follows: (1) government leadership; (2) PE curriculum; (3) school principal and leaders; (4) organisational management from leadership in school; (5) teachers; (6) parental involvement; and (7) community partnerships. Based on these findings, recommendations were made for an evaluation framework on QPE in primary education.

## 1. Introduction

Quality physical education (QPE) can be explained as a group of interrelated strategies to embrace the formulation and development of inclusive and equitable curricula. It provides personally meaningful as well as socially and culturally relevant experiences. These attract young people to the joy and pleasure of physical activity (PA) to foster an active healthy lifestyle over their full lifespan [1]. Children have the right to QPE and should have equal access to qualified professionals, appropriate resources and supportive structures regardless of their background. Schools are the medium to provide QPE and represent an attractive and important setting for health promotion initiatives [2]. Based on the significant amount of time children spend at school, this sector has a great influence on promoting and improving PA in youth [3]. Worldwide, PE is by far the most common method of promoting PA during the school day, and the majority of countries have legal requirements for school PE for at least some part of the compulsory schooling years. Even in countries where PE may not be mandated by law, the subject is still offered [3].

QPE consistently receives positive acclaim as a contributor to young learners’ holistic development, including academic achievement (AA), mental wellbeing (MWB) and PA, especially in their formative years [4]. Research on children’s and adolescents’ PA levels shows that their overall PA levels tend to be low and decline with age [5,6,7]. Physical inactivity is recognised as a public health issue across all ages. PA in children is associated with improvements in skeletal health, CVD risk factors, adiposity, self-esteem and MWB [8]. As such, it is essential to continue the research on the factors that contribute to or inhibit children from engaging with PA in its different forms, including PE, to minimise their sedentary lifestyle and reap all the PA benefits that will contribute to a fuller, longer, better and happier life [9]. Consistent with the notion of a link between early-life experiences and later health outcomes, greater attention is being given to the importance of exposure to QPE opportunities during early years and childhood. If health behaviours established during early life are more likely to persist or ‘track’ from childhood to adulthood, greater efforts should be made to capitalise on key opportunities, including in the school setting [3]. PE is a fundamental cornerstone for childhood development as it promotes lifelong PA participation for holistic health. School educators play a key role in creating school environments that lead to developmentally appropriate and high-quality PE lessons [10]. QPE opportunities should be carefully considered so that children will develop lifelong habits of being active, as well as create positive attitudes towards PE (ATPE). It is imperative that school leaders identify the key features of QPE programmes that help promote all the outcomes on ATPE, PA behaviour (PAB), AA and MWB. This study aims to scope out these key features.

Systematic reviews have identified over 100 randomised trials of school-based interventions targeting PA among other health-related factors. They have demonstrated that such interventions can be effective in reducing associated health risks [11]. Based on this evidence, national and international best-practice guidelines have been established acknowledging the potential for school-based settings to influence the development of children’s PAB. These evidence-based guidelines recommend schools adopt a range of policies, practices and programmes, such as the scheduling and provision of QPE programmes, and active play opportunities [12].

In Barne’s systematic review, the findings provide some evidence to support the effectiveness of strategies in enhancing the nutritional quality of foods served at schools, the implementation of canteen policies and the time scheduled for physical education [13]. Advocacy needs to be directed toward the school officials and community members. There are approximately 25 million obese children in the United States of America (USA) today, and that number continues to grow. Schools that are reducing or cutting their PE programmes are limiting the students’ access to healthy eating and fitness [14].

Other reviews have found that enhancing the quality of policy dissemination and implementation research through high-quality pragmatic measures will mark a key step in bridging the policy-to-practice gap [15]. Policy research over the last 20 years was focused on the regulatory mandate of time. Policy research did not directly address disciplinary process variables of learning activities or outcomes of physical education. The effects of policy exemptions and class size were underrepresented. Further research is needed to examine the downstream effects of physical education policy and determine whether well-written policies increase the number of individuals that are more physically competent [16].

The interest in the connection between PE programmes and AA during development has recently increased, with evidence suggesting a positive association [17]. This is very encouraging, as part of this study will attempt to examine the impact of QPE programmes on pupils’ AA as well as on ATPE, PAB and MWB. To assess this impact, the implementation fidelity of QPE programmes will be initially investigated. To ensure the integrity of scientifically based research, curriculum intervention researchers conducting efficacy and effectiveness studies are now being asked to describe how fidelity is defined, conceptualised and measured. Fidelity measures are needed to explain the degree of variation in implementation and how this might affect moderate outcomes. This means that fidelity measures must capture the critical features of the intervention’s programme theory [18]. Such measures should also indicate how the intervention is maintained consistently across multiple groups over time or describe the parameters under which variations in the implementation may occur. The statistical power of a study relies on the use of reliable, valid measures; appropriate design and sampling; and careful assessment of fidelity. Each of these three decreases as research moves from the laboratory (efficacy studies) and toward the field (effectiveness studies) [19]. Therefore, measuring the fidelity of implementation is critical to determine not only if the QPE programme is sufficiently implemented, but also if there are critical differences between what the experimental and comparison groups receive, so that one might expect a difference in outcomes.

While the reviews mentioned above have presented many findings, there are still gaps in the research. Therefore, this review aims to scope out the common themes of features of QPE programmes in primary schools, while considering the four outcome dimensions (i.e., ATPE, PAB, MWB and AA). The aim of the review is to formulate an evaluation framework on QPE in primary education.

## 2. Materials and Methods

A scoping review of the available qualitative and quantitative literature relating to the evaluation of implementation fidelity of quality QPE programmes and their outcomes on final-stage primary-level pupils’ ATPE, PAB, MWB and AA, was conducted according to the PRISMA extension for scoping reviews guidelines (PRISMA-ScR) [20]. Using the PRISMA-ScR guidelines of identification, screening, determining eligibility and inclusion, studies were extracted from four different databases, using search terms related to the essential PE component of policy and environment.

### 2.1. Inclusion and Exclusion Criteria

The inclusion criteria were as follows: (1) studies that explored the attitudes of pupils on PE, as well as outcomes on AA, PAB and MWB (outcome criteria); (2) studies that used qualitative and quantitative methods to collect data (design criteria); (3) pupils aged between 8 and 12 years, from urban and non-urban areas living in developed or developing countries (population criteria); (4) studies published in English (language criteria); (5) studies published after 2000 (time criteria); (6) studies published in scientific journals (publish criteria).

Studies were excluded if they (1) were not focused on PAB outcomes, ATPE outcomes, MWB outcomes and AA outcomes; (2) focused on out-of-school PA; (3) were a systematic review themselves; (4) involved participants over 12 years; (5) were not published in English; (6) were published before 2000; and (7) were not articles published in scientific journals with peer review (e.g., conference papers, book chapters, etc.).

### 2.2. Search Strategy

Regarding the search strategy, PubMed, Elsevier, SCOPUS and CINAHL databases were used to ensure, from an early stage, the scientific quality of the studies ranging the areas of social science and health sciences that were core to this research. The search strategy was based on the following fields: ‘title’, ‘abstract’ and ‘keywords/subject’. The language of publication was restricted to English. The terms used in the search were: ‘quality physical education*’ AND ‘physical education programme’ AND ‘primary*’ AND ‘education’. Additional records were identified through reference lists. In those cases where the scientific article or data needed were not available (e.g., no access to pdf, no mean age, etc.), the authors of the study were contacted via email and/or professional media platforms.

### 2.3. Studies’ Screening, Selection and Quality

After performing the search in the databases, the data were imported into a reference manager software [21]. One hundred fifty-two duplicate studies were eliminated automatically. The first author screened titles and abstracts of the remaining records. Each full-text article was carefully examined to decide whether the article met the inclusion criteria and, if so, to assess its quality. Where there were doubts, these were discussed with the research team and an agreement was reached upon inclusion or exclusion of the reference.

**Figure 1:**
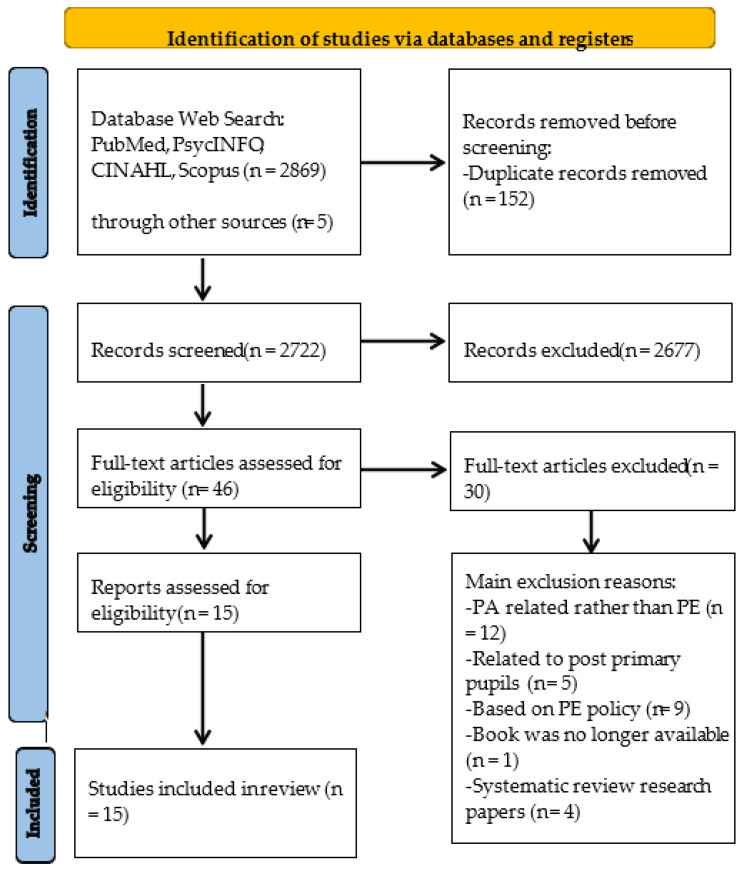
PRISMA-ScR flow diagram for study selection.

The initial search identified 2869 records in the described databases, and an additional 5 records were identified through the reference list. These data were then exported to the reference manager software EndNote and all duplicates (152 records) were excluded. The remaining 2722 studies were then screened according to the title and abstract for relevance, resulting in another 2677 studies being eliminated from the database. The full texts of the remaining 46 studies were read in full and another 30 were further excluded due to lack of relevance for the specific purpose of the current review. The main reasons for exclusion were related to the following: (1) PA-related rather than PE (n = 12); (2) related to post-primary pupils (n = 5); (3) based on PE policy (n = 9); (4) book was no longer available (n = 1); and (5) systematic review research papers (n = 4). At the end of the screening procedure, 15 studies were included. See Figure 1 below.

### 2.4. Data Extraction, Analysis and Synthesis

Initially, each article was read, and the following characteristics were extracted independently by the first author of the scoping review: (1) author’s name and publication year; (2) country; (3) sample; (4) methods; (5) quality feature of PE programme; (6) dimension of outcome; (7) physical measurement for each outcome; and (8) main findings. Data extracted by the researcher were re-examined, readjusted and confirmed with three other authors. The four outcome dimensions were considered as deductive categories: ATPE, PAB, MWB and AA. Following this, a thematic synthesis approach was adopted to analyse and synthesise the data concerning the research topic. In this inductive/deductive process, each article was read several times and analysed line by line by the first author. The features were constantly compared, and then coded according to the thematic emphasis in the seven selected themes. The full coding was reviewed between the four members of the research theme involved in the process and confirmed after reviewing and refining the coding scheme.

## 3. Results

### 3.1. Study Characteristics

Table S1 (Supplementary File) presents the characteristics of the studies included in this systematic review between 2000 and 2020. For this review, the perspectives of researchers from nine countries were taken into account. Three of the included studies were carried out in Australia, three in Malaysia, two in Ireland, two in the USA and one in each one of the following countries: United Kingdom, Greece, Italy, Spain and South Africa. Studies were cross-sectional: five were qualitative, seven used a mixed-methods approach and three studies were quantitative in nature. In relation to the dimension outcomes, fourteen studies reported on the dimension ATPE, four on the dimension PAB, one on the dimension MWB and one on the dimension AA. As for the implementation fidelity of a QPE programme and its outcomes on ATPE, PAB, MWB and AA, 12 out of 15 studies did not directly relate to this; however, all studies were related to the topic of implementation fidelity indirectly. Details were provided on the sample of each study. The specific method was outlined, and several quality features were inductively and deductively identified across all four dimensions of the study. The physical measurement for each dimension was also outlined along with the main findings (Table S1).

### 3.2. Domains of QPE in Primary Education

Following further analysis, all studies were categorised into seven themes summarising the domains of QPE programmes in primary education that have shown to positively affect one or more of the outcome dimensions targeted by this study (ATPE, PAB, MWB and AA). The following themes were generated: (1) government leadership; (2) PE curriculum; (3) school principal and leaders; (4) organisational management from leadership in school; (5) teachers; (6) parental involvement; and (7) community partnerships. Each theme was composed of the specific features of quality (Table 1). Teachers (Theme 5) were by far the most heavily weighted theme with the largest number of associated quality features.

#### 3.2.1. Theme 1: Government Leadership

Through the scoping review, five studies were linked to the theme government leadership. These included five studies [3,22,23,24,25]. The selected studies positively affected two of the dimensions: ATPE and AA. It is imperative that governments display leadership by enabling schools to establish health-promoting environments that support PA [3]. It was found that adequate government leadership was associated with the provision of the adequate availability of PE facilities such as open fields, outdoor obstacle courses and playgrounds [22]. Leaders could provide this by building new facilities or upgrading existing ones. Training educators on the use and maintenance of facilities were also important [23]. Teachers reported that a good level of resources provided were invaluable in supporting and enabling their PE teaching [24]. Equipment availability, quality of facilities, level of departmental assistance, class size and access to professional development were key features linked to good government leadership [25].

#### 3.2.2. Theme 2: PE Curriculum

The theme PE curriculum aligned with five of the studies from the scoping review [26,27,28,29,30]. They were found to affect only one of the outcome dimensions, namely ATPE. Lucertini et al. [26] reported a number of features that enhanced the PE curriculum. The study reported the following: (1) specialist-led lessons benefitted pupils more than generalist-led peers. Specialist-led pupils demonstrated greater increases in some motor and health-related abilities tests compared to generalist-led peers; (2) 60-minute PE lessons were more beneficial than shorter lessons; and finally, (3) structured programmes were more effective than unstructured. Other features that were reported to be beneficial were fundamental movement skills and curriculum flexibility [27]. Similar to Lucertini et al. [26], it was suggested that a structured curriculum to help acquire skills, knowledge and dispositions was necessary to be ‘wise consumers’ of PA [28]. The positioning of PE in the curriculum [24] and curriculum management-planning and implementation [30] were also noted in the scoping review as key features of this theme.

#### 3.2.3. Theme 3: School Principal and Leaders

Both Wee [24] and Fonyi and Soon [31] are closely linked with this theme. The studies affected only one outcome dimension: ATPE. There were a number of quality features observed in this study which were associated with school principals and leaders. Features worth noting were principals’ positioning of PE in the curriculum; adequate funding for the PE department from school administrators; administrators discussing PE teaching assignment with teachers; PE classes assigned to teachers based on their professional qualification, leadership and vision (status of PE as a subject); and adequate library PE books [29].

The academic and professional qualifications of the principal were also important. When principals believed in personal development, they would also support the welfare of teachers to ensure quality PE learning and teaching experiences. The school context and years of teaching experience were relevant. Promoting a positive and quality school learning climate was directly influenced by what a principal had gone through in their lifetime as a teacher. The PA level of school principals was influential, and principals who were involved in organised PA were more likely to possess a higher favourable attitude towards administering QPE programmes as compared to less-active principals [31]. This study also stated that both female and male principals favoured PE and believed in the importance of it. All of the above features were closely connected to the successful implementation of QPE programmes in schools.

#### 3.2.4. Theme 4: Organisational Management from Leadership in School

Quality features for this theme can be seen in the study by Kulik, 2009, [22]. The only associated outcome dimension was ATPE. The following quality features were identified under the theme organisational management from leadership in school: meeting the needs of the staff; providing readiness factors such as equipment and sports facilities, re-sources and materials; provision of multiple PE opportunities (recess, intramurals, interscholastic sports, classroom PA breaks and walk and bicycle to school initiatives); and promoting extracurricular physical activity. PA before, during and after the school day, and multiple opportunities for physical activity throughout the school day were also important [22].

#### 3.2.5. Theme 5: Teachers

There was a wide selection of quality features identified under this theme. It is worth noting that many of these features overlapped in the 15 studies reviewed. The quality features for this theme were found in the following studies [3,22,23,24,25,27,28,29,30,32,33,34,35]. Of all seven themes, teachers were by far the most heavily weighted theme. It is interesting to note that this was the only theme that affected all four outcome dimensions: ATPE, PAB, MWB and AA.

The key features included personal school experiences in PE for teachers, years of teaching experience, teachers’ confidence and competence for teaching PE, teachers’ interest/enthusiasm for PE, willingness to teach new elements of PE content and attitudes to meeting the individual needs of children in PE. Additionally relevant were teachers serving as the school PA leader, planning and implementing and cooperative learning opportunities, providing goal-oriented activities, quality preparation of tutors, communities of practice and collaborative learning. Specialist-led teachers, teacher education, observations of other teachers’ PE classes, providing students with learning experiences that meet individual developmental needs, being knowledgeable about the PE curriculum, unpacking its specialised content, using available resources appropriately and designing suitable learning programmes were also identified as quality features.

#### 3.2.6. Theme 6: Parental Involvement

Features included in this theme were identified mainly in the Christodoulos et al. [32] study. This theme affected just one outcome dimension: PAB. The features included parental support for the PE programme, positive ATPE, support for school transport initiatives, assisting the school to deliver the PE programme and fundraising and helping to advance the PE facilities and resources in the school. Parental involvement was encouraged through homework assignments with family activities, by sending educational material home, providing physical activity and nutritional guidelines and by asking parents to send healthy snacks to school.

#### 3.2.7. Theme 7: Community Partnerships

The final theme can be associated with three studies from the scoping review (Christodoulos et al. [32], Hills et al. [3] and Lynch et al. [27]). By promoting extracurricular PA and disseminating information about community-based sports programmes, QPE in schools can be strengthened [32]. Family and community involvement in PA and PE programmes in schools was worthwhile [3]. There were also opportunities for community partnerships so that quality PA resources could be provided through building new facilities and upgrading existing ones [27]. All features listed above were important so that QPE programmes could be delivered successfully in schools.

## 4. Discussion

This scoping review updates the existing knowledge from quantitative and qualitative research relating to the evaluation of the implementation fidelity of QPE programmes and their outcomes on final-stage primary-level pupils’ ATPE, PAB, MWB and AA. In this section, the quality features are organised and discussed around the four outcome dimensions (A1). At the end, strengths, recommendations and limitations are further developed.

### 4.1. Attitudes towards Physical Education

School PE provides a context for regular and structured PA participation. To this end, a common justification for PE’s place in the school curriculum is that it contributes to children’s health and fitness [36]. Christodoulos et al. [32] reported that the higher scores of the intervention classes on attitudinal measures, compared with the typical classes, can be attributed to the health-related lectures and frequent health-related reminders included in the teaching material. For example, when students were doing sit ups, the teacher could remark ‘our abdominals help in good posture’; when they were running, the teacher could remark that ‘now we are improving aerobic endurance’. There were reminders for the PE teacher to give as many such messages as possible. This approach may be beneficial in attitude change. The Christodoulos et al. [32] study also focused on children’s enjoyment and willingness to participate in PE classes. Christodoulos et al. [32] suggested that emphasis should be placed on total class participation in enjoyable, non-competitive exercise forms. PAs matched to the child’s ability are more likely to produce a feeling of success than those that are at too high a skill level. Another perspective is that students believe their peers play a major role in impacting their attitudes towards PE, but research has demonstrated that the influences of the PE teacher and curriculum are more powerful in this regard [37,38]. Previous studies have shown that the involvement of parents remains a necessary part of school-based PE programmes, because their attitude has a great influence on the lifestyle and habits of health behaviour in children [39]. There is an obvious link between the dimension ATPE and the quality features of the PE curriculum, teachers and parental involvement. As a result, it is suggested to include health-related information as part of the PE curriculum content, and that teachers be properly trained so that adequate and well-structured content is delivered. It is also important for parents to be involved in some aspects of the delivery of the PE programme in what concerns the promotion of positive attitudes.

It was reported that specialised teachers are more impactful in the classroom as opposed to generalised teachers [26], and that PE specialists provide more effective PE than non-specialists [40]. Another experiment showed that the planning and delivery of PE lessons by PE-specialist teachers in comparison to generalist teachers resulted in a relative improvement of physical fitness in the quasi-test group in comparison to the quasi-control group [41].

A major barrier to delivering QPE programmes is the poor qualifications and preparation of teachers. It is recommended that opportunities for developmentally appropriate primary education PE specialism be provided within degrees, allowing every primary school over time to have a sustainable infrastructure of PE expertise and advocacy [27]. Similar to the point made by Lucertini et al. [26] and supporting the point made by Lynch et al. [27], there is a need to have specialist PE teachers, as the majority of the current teachers taught less than five PE periods per week and only 6.2% were PE majors [29]. The above results make evident the connection between ATPE and the domains government leadership and teachers regarding teacher preparation, qualification and professional development. The government should consider the impact of the academic and professional background of those who are in charge of delivering successful PE programmes in schools, and should prioritise the contribution, or collaboration, of specialist PE teachers in primary education systems where the delivery of the PE subject is the responsibility of generalist teachers.

Time allocation is another relevant indicator of QPE programmes for ATPE. There is evidence to support the effectiveness of PE policy strategies in enhancing the time scheduled for PE [42]. It is reported that schools in the USA do not offer PE daily and also do not offer the recommended 225 min (45 min per day × 5 days per week) of PE per week [42]. It does not appear that the factors recommended by the Society of Health and Physical Educators (SHAPE) America influence the amount of time that is allocated for PE instruction [22].

Administrators also play a major role in ensuring high-quality PE implementation, which highlights the strong links between ATPE and the domains of school leaders and organisational management from leaders in school. In a systematic review carried out by McLoughlin et al. [43], one of the higher implementation determinants assessed was leadership (n = 42, 49%) [43]. The teachers need to emphasise the importance of PE and value it similar to other subjects. The administrators should strive to ensure that the PE curriculum is properly pursued by assigning it its due budget and allocating proper resources so that activities can be carried out in accordance with the syllabus. The lack of resources for PE activities results in a general lack of enthusiasm among students for PE [30]. Good administrators organise in-house training, discuss PE teaching assignments and factors affecting PE teaching and observe PE teaching. As regards class distribution, administrators should have discussions with teachers before assignment to ensure PE teaching is based on interest and qualification [29].

It is evident that certain features are prerequisites for QPE programmes. The information gathered from this scoping review highlights that the key features that positively affect ATPE are health-related reminders, foci on children’s enjoyment and willingness to participate in PE classes, specialised teachers, adequate time allocation and administrators working to ensure that the PE curriculum is properly pursued. The effectiveness of these key features depends on the success of the following domains: government leadership, PE curriculum, school principal and leaders, organizational management from leadership and teachers. All these messages along with their obvious links to the domains listed above should be taken into account in the design of future QPE interventions aimed at improving ATPE in children.

### 4.2. Physical Activity Behaviour

The foundations of PAB are set early in life, and schools have an important role to play in shaping young people’s activity behaviours. PE is meant to teach students the benefits of health and exercise not only for the duration that they are in school, but for their lifetime. Schools without a quality health or PE programme are inevitably pushing young students toward a sedentary adult lifestyle [14]. A comprehensive school physical activity programme (CSPAP) should consist of PE and other PA opportunities such as recess, intramurals, interscholastic sports, classroom PA breaks as well as walking and cycling to school initiatives [44]. The crowded school curriculum with an intense focus on AA; lack of school leadership support, funding and resources; along with poor-quality teaching are barriers to QPE and PA promotion in schools [3]. Due to the relationship schools have with their communities, both local and national governing bodies need to be involved in developing CSPAPs. This supports the connection between PAB and government leadership. Governments are able to support leadership by requiring schools to provide PE and other PA opportunities before, during and after school and by enabling schools to establish health-promoting environments that support PA [45].

Data show that school health education programmes, including PE programmes, have the potential to slow the age-related decline in PA and help pupils establish lifelong, healthy PA patterns. Promoting healthy habits and PA behaviours during childhood may prevent some of the leading causes of morbidity and mortality, while also decreasing direct healthcare costs and improving quality of life [32], thus highlighting the link between PAB with the domains of school principal and leaders, and teachers. It is imperative that school leaders and PE teachers address this deficit and ensure that the minimum requirement of allocated time for PE is met.

A final connection became evident between the dimension PAB and the domains of teachers and parental involvement. Physical educators must be key drivers of physical and health literacy as well as behaviour changes to optimise the QPE of children and adolescents. For parents, physical educators need to provide information regarding the benefits of PA via messages sent home and different school activities. In addition, physical educators need to encourage families to become involved in school-based PA and events. Since non-specialist classroom teachers and staff often serve as role models for children, physical educators need to encourage school staff to be more physically active [3].

### 4.3. Mental Wellbeing

Cooperative learning as a pedagogical model that can contribute to the students’ social and emotional learning involves building quality relationships, learning to manage stressors and evolving as individuals and as a group. The domain teachers links closely to this dimension (i.e., MWB) as teachers can plan and provide cooperative learning opportunities for children in school. The cooperative learning framework helps to increase students’ self-approach goals and their emotional control and regulation, and empathy. It can guide students to more adaptive motivational patterns and to develop their emotional intelligence [35]. QPE programmes are well-positioned to include cooperative learning activities and are therefore significant factors in determining positive outcomes for pupils’ wellbeing.

### 4.4. Academic Achievement

International research in this field has repeatedly reported that increasing PA can be positively related to AA. It has been proven that when students are active for a portion of their day, they are able to focus and be more productive in school [46]. Physical educators now have data showing that PA can help students learn better and releases endorphins that improve mood [14]. Higher cardiorespiratory fitness may be important to enhance children’s and adolescents’ health, and, additionally, AA [47]. Due to a lack of consensus across studies, methodological issues associated with the assessment of QPE and PA should be considered when investigating the links between PE/PA and AA. QPE and PA seem not to be detrimental to school-age children and adolescents’ AA, and may, in fact, be beneficial [48]. The Department of Education in America explicitly lists the personal development, health and PE (PDHPE) key learning area (KLA) as how schools support and develop students’ health, emphasising physical and mental health, as well as academic benefits [27]. Further research shows that participating in PE is positively related to outcomes including academic achievement; academic behaviors; and indicators of cognitive skills and attitudes, such as concentration, memory, self-esteem and verbal skills [46]. All studies reviewed emphasise the link between the AA dimension and government leadership. It is crucial that governments continue to invest in researching the links between AA, PAB and ATPE as interconnected variables that contribute to the pupils’ overall wellbeing [49].

## 5. Conclusions

A key strength of this scoping review is that it is the first to evaluate the literature on the implementation fidelity of QPE programmes and their outcomes on final-stage primary-level pupils’ ATPE, PAB, MWB and AA. Furthermore, the concurrent examination of research on features of QPE programmes and their outcomes on final-stage primary-level pupils’ ATPE, PAB, MWB and AA enabled a comprehensive investigation of the most up-to-date evidence. The focus on peer-reviewed research enhanced the overall integrity and rigor of this scoping review and strengthened the review’s methodological quality. Reliance on empirical studies that analyse primary data, complemented with additional sources of evidence (e.g., different types of reviews, scientific statements, position papers), is another clear strength of our review because we are able to provide a holistic and more nuanced view of the existing evidence for the evaluation of the implementation fidelity of QPE programmes and their outcomes on final-stage primary-level pupils’ ATPE, PAB, MWB and AA. This is one of a few scoping reviews that is used to identify quality features of PE programmes and to use these to develop a conceptual framework. Furthermore, the key features of QPE programmes that have been identified can be used to inform future researchers, PE policy makers and PE teachers. There is an obvious link between the four outcome dimensions and the seven identified domains displayed in Table 1. The findings indicate that domain 5 (teachers) has far more quality features than any of the other six. This gives us evidence that teachers are a highly significant and a very valuable resource when discussing the topic of QPE programs in primary schools. More emphasis should be placed on researching this body of people and considering the subsequent findings.

However, there were some limitations, with the main one being the quality of analysed research was not examined because the primary aim of this review was to identify the existing features of QPE programmes that promote one or more of the four outcome variables. However, we acknowledge that a quality evaluation is needed for similar studies which primarily focus on the analysis of the outcomes. Another limitation is that this review did not aim to analyse the outcomes themselves; therefore, an analysis of the impact of the features of QPE programmes on the outcome variables is needed.

An important conclusion from this scoping review lies in the volume of available studies. There is limited and disperse research completed in this area; therefore, the number of relevant studies selected to be analysed in relation to each outcome variable was reduced. Another conclusion from this review is that there were no longitudinal studies as none were found during the database search. This compromises the existing knowledge based on the long-term effects of QPE programmes and their outcomes on pupils’ ATPE, PAB, MWB and AA in primary school settings. This study recommends that further research is needed, and particularly longitudinal studies towards a more concentrated effort in researching in this area. It was consistent throughout this study that studies relating to the effect of QPE programmes on the MWB and AA of pupils in primary schools were less available than the effects on any of the ATPE and PAB outcome dimensions. Only one paper was selected for the topic MWB and one for AA as part of this scoping review. Policy makers and researchers alike should consider a broad and balanced approach to monitoring the longitudinal impact of the fidelity of implementation of QPE programmes on the four outcome variables that were addressed in this study for a holistic picture of children’s development at the end of their primary education.

## Figures and Tables

**Table 1:** Themes and quality features identified across all four dimensions in the systematic review.

Themes and Quality Features Identified
Government leadership Facilities, equipment and resources; Human resources; Availability of PE facilities: facilities such as open fields, outdoor obstacle course, playground equipment; Quality physical resources through building new facilities and upgrading existing ones; Train educators on the use and maintenance of facilities; Class size; Level of departmental assistance. PE curriculum Fundamental movement skills; Curriculum flexibility; Structured curriculum to help acquire skills, knowledge; Reflection on the nature and content of programmes of PE; Time—60 min or more of moderate-intensity physical activity each day; Instruction time—45 min per day × 5 days per week = 225 min per week. School principal and leaders Adequate funding allocated to the PE department; Administrators discussing PE teaching assignments with teachers; Academic and professional qualifications of the principal; Personal school experiences in PE of the principal; PE classes assigned to teachers based on their professional qualification; Adequate library PE books; Equipment availability; Quality of facilities; School executive attitudes towards PE; Leadership and vision: status of PE as a subject. Organisational management from leadership in school Needs of the staff, readiness factors such as equipment and sports facilities; Resources and materials; Multiple PE opportunities—recess, intramurals, interscholastic sports, classroom PA breaks and walk and bicycle to school initiatives; Promote extracurricular physical activity; PA during the school day, PA before and after school; Multiple opportunities for physical activity throughout the school day. Teachers PE content knowledge; Personal school experiences in PE for teachers; Years of teaching experience; Teacher’s confidence teaching PE; Teacher’s interest/enthusiasm for PE; Willingness to teach new elements of PE content; Attitudes to meeting the individual needs of children in PE; Teachers serving as the school physical activity leader; Planning and implementation; Cooperative learning opportunities; Goal-oriented activities; Quality preparation of tutors; Teachers’ attitudes; Communities of practice and collaborative learning; Specialist-led teachers; Teacher competency; Teacher education; Observations of other teachers’ PE classes; Providing students with learning experiences that meet individual developmental needs, which help to improve mental alertness, academic performance, readiness to learn and enthusiasm for learning; Teacher to be knowledgeable about the school curriculum, unpack its specialised content, use available resources appropriately and design suitable learning programmes. Parental involvement Support for the PE programme; Positive attitude towards PE; Support for school transport initiatives; Assisting school to deliver the PE programme; Fundraising and helping to advance the PE facilities and resources in the school; Homework assignments with family activities; Sending educational material home; Providing physical activity and nutritional guidelines; Asking parents to send healthy snacks to school. Community partnerships Promoting extracurricular physical activity and disseminating information about community-based sports programmes; Family and community involvement in PA and PE programmes in schools; Community partnerships so that quality physical resources can be provided through building new facilities and upgrading existing ones.

## Data Availability

Not applicable.

## Supplementary Materials

The following supporting information can be downloaded at: https://www.mdpi.com/article/10.3390/ijerph20043575/s1. Table S1: Characteristics of the included studies in the scoping review.

## References

[1] McLennan N., Thompson J. (2015). Quality Physical Education (QPE): Guidelines for Policy Makers.

[2] World Health Organization (2017). Report of the Commission on Ending Childhood Obesity.

[3] Hills A.P., Dengel D.R., Lubans D.R. (2015). Supporting Public Health Priorities: Recommendations for Physical Education and Physical Activity Promotion in Schools. Prog. Cardiovasc. Dis..

[4] Jess M., Carse N., Keay J. (2017). The primary teacher, professional development and physical education. Routledge Handbook of Primary Physical Education.

[5] Guthold R., Stevens G.A., Riley L.M., Bull F.C. (2020). Global trends in insufficient physical activity among adolescents: A pooled analysis of 298 population-based surveys with 1·6 million participants. Lancet Child Adolesc. Health.

[6] Marques A., Henriques-Neto D., Peralta M., Martins J., Demetriou Y., Schönbach D.M.I., De Matos M.G. (2020). Prevalence of Physical Activity among Adolescents from 105 Low, Middle, and High-Income Countries. Int. J. Environ. Res. Public Health.

[7] Sallis J.F., Bull F., Guthold R., Heath G.W., Inoue S., Kelly P., Oyeyemi A.L., Perez L.G., Richards J., Hallal P.C. (2016). Progress in physical activity over the Olympic quadrennium. Lancet.

[8] Biddle S., Gorely T., Stensel D. (2004). Health-enhancing physical activity and sedentary behaviour in children and adolescents. J. Sports Sci..

[9] Martins J., Costa J., Sarmento H., Marques A., Farias C., Onofre M., Valeiro M.G. (2021). Adolescents’ Perspectives on the Barriers and Facilitators of Physical Activity: An Updated Systematic Review of Qualitative Studies. Int. J. Environ. Res. Public Health.

[10] Donnelly F.C., Mueller S.S., Gallahue D.L. (2016). Developmental Physical Education for All Children: Theory into Practice.

[11] Demetriou Y., Höner O. (2012). Physical activity interventions in the school setting: A systematic review. Psychol. Sport Exerc..

[12] Government of Canada (2019). Healthy Eating at School. https://food-guide.canada.ca/en/tips-for-healthy-eating/school/.

[13] Barnes C., McCrabb S., Stacey F., Nathan N., Yoong S.L., Grady A., Sutherland R., Hodder R., Innes-Hughes C., Davies M. (2021). Improving implementation of school-based healthy eating and physical activity policies, practices, and programs: A systematic review. Transl. Behav. Med..

[14] (2015). How do we best advocate for quality health and physical education in an economically challenging school climate?. J. Phys. Educ. Recreat. Dance.

[15] Glasgow R.E., Riley W.T. (2013). Pragmatic measures: What they are and why we need them. Am. J. Prev. Med..

[16] Burson S.L., Mulhearn S.C., Castelli D.M., van der Mars H. (2021). Essential Components of Physical Education: Policy and Environment. Res. Q. Exerc. Sport.

[17] Corredor C. (2015). The Effects of Physical Education on Academic Achievement; Applied Research in Children’s Studies, Children and Youth Studies.

[18] Education C., Bruton G., Accountant I. (2006). National Center for Education Statistics.

[19] Mowbray C.T., Holter M.C., Teague G.B., Bybee B. (2003). Fidelity Criteria: Development, Measurement, and Validation. Am. J. Evaluation.

[20] Tricco A.C., Lillie E., Zarin W., O’Brien K.K., Colquhoun H., Levac D., Moher D., Peters M.D., Horsley T., Weeks L. (2018). PRISMA extension for scoping reviews (PRISMA-ScR): Check-list and explanation. Ann. Intern. Med..

[21] Gotschall T. (2021). EndNote 20 desktop version. J. Med. Libr. Assoc..

[22] Kulik K.S. (2001). Implementation of a Quality Physical Education Program as Defined by the National Association for Sport and Physical Education of Public High Schools in Southern Pennsylvania.

[23] Roux K.C.J. (2020). The delivery of primary school physical education in South African public schools: The perceptions of educators. S. Afr. J. Child. Educ..

[24] Coulter M., Woods C.B. (2012). Primary teachers’ experience of a physical education professional development programme. Ir. Educ. Stud..

[25] Morgan P.J., Hansen V. (2008). Classroom teachers’ perceptions of the impact of barriers to teaching physical education on the quality of physical education programs. Res. Q. Exerc. Sport.

[26] Lucertini F., Spazzafumo L., De Lillo F., Centonze D., Valentini M., Federici A. (2013). Effectiveness of professionally-guided physical education on fitness outcomes of primary school children. Eur. J. Sport Sci..

[27] Lynch T., Soukup Sr G.J. (2017). Primary physical education (PE): School leader perceptions about classroom teacher quality implementation. Cogent Educ..

[28] Tyler G., Turner J.L. (2016). The Physical Activity Movement and the Definition of Physical Education. J. Phys. Educ. Recreat. Dance.

[29] Wee E.H. (2019). Social Sciences and Humanities Implementation of Physical Education Pro-gramme in Malaysian Primary Schools. Pertanika J. Soc. Sci. Humanit..

[30] Mohamed A.M.D., Amri S., Kok L.Y., Abdullah B. (2017). Factors influencing the implementation level of physical education in Selangor primary schools. Int. J. Acad. Re-Search Bus. Soc. Sci..

[31] Fonyi L., Soon C.C. (2021). Administrators’ Attitude towards the Implementation of Physical Education in Selangor Primary Schools. Pertanika J. Soc. Sci. Humanit..

[32] Christodoulos A.D., Douda H.T., Polykratis M., Tokmakidis S.P. (2006). Attitudes towards exercise and physical activity behaviours in Greek schoolchildren after a yearlong health education intervention. Br. J. Sports Med..

[33] Murphy F., O’Leary M. (2012). Supporting primary teachers to teach physical education: Continuing the journey. Ir. Educ. Stud..

[34] Atencio M., Jess M., Dewar K. (2012). It is a case of changing your thought processes, the way you actually teach: Implementing a complex professional learning agenda in Scottish physical education. Phys. Educ. Sport Pedagog..

[35] Rivera-Pérez S., Fernandez-Rio J., Gallego D.I. (2020). Effects of an 8-Week Cooperative Learning Intervention on Physical Education Students’ Task and Self-Approach Goals, and Emotional Intelligence. Int. J. Environ. Res. Public Health.

[36] Physical Education Association of the United Kingdom (2004) PEA UK Policy on the Physical Education Curriculum. http://www.pea.uk.com/menu.html.

[37] Figley G.E. (1985). Determinants of attitudes towards physical education. J. Teach. Phys. Educ..

[38] Luke M.D., Sinclair G.D. (1991). Gender differences in adolescents’ attitudes towards physical education. J. Teach. Phys. Educ..

[39] Dobbins M., Husson H., DeCorby K., LaRocca R.L. (2013). School-based physical activity programs for promoting physical activity and fitness in children and adolescents aged 6 to 18. Cochrane Database Syst. Rev..

[40] Starc G., Strel J. (2012). Influence of the quality implementation of a physical education curriculum on the physical development and physical fitness of children. BMC Public Health.

[41] Kriemler S., Zahner L., Schindler C., Meyer U., Hartmann T., Hebestreit H., Brunner-La Rocca H.P., van Mechelen W., Puder J.J. (2010). Effect of school based physical activity programme (KISS) on fitness and adiposity in primary schoolchildren: Cluster randomised controlled trial. BMJ.

[42] Nathan N., Wiggers J., Bauman A.E., Rissel C., Searles A., Reeves P., Oldmeadow C., Naylor P.J., Cradock A.L., Sutherland R. (2019). A cluster randomised controlled trial of an intervention to increase the implementation of school physical activity policies and guidelines: Study protocol for the physically active children in education (PACE) study. BMC Public Health.

[43] McLoughlin G.M., Allen P., Walsh-Bailey C., Brownson R.C. (2021). A systematic review of school health policy measurement tools: Implementation determinants and outcomes. Implement. Sci. Commun..

[44] Wechsler H., McKenna M.L., Lee S.M., Dietz W.H. (2004). The role of schools in preventing childhood obesity. State Educ. Stand..

[45] SHAPE America (2017). National Standards for Initial Physical Education Teacher Education.

[46] Centers for Disease Control and Prevention (2010). The Association between School Based Physical Activity, Including Physical Education, and Academic Performance.

[47] Marques A., Santos D.A., Hillman C.H., Sardinha L.B. (2018). How does academic achievement relate to cardiorespiratory fitness, self-reported physical activity and objectively reported physical activity: A systematic review in children and adolescents aged 6–18 years. Br. J. Sport. Med..

[48] Barbosa A., Whiting S., Simmonds P., Moreno R.S., Mendes R., Breda J. (2020). Physical Activity and Academic Achievement: An Umbrella Review. Int. J. Environ. Res. Public Health.

[49] National Council for Curriculum and Assessment and the Department of Education and Skills, Republic of Ireland; Annual Report 2017. https://ncca.ie/en/resources/ncca-annual-report-2017/.

