# Supplementary material for: A Scoping Review on Quality Physical Education Programmes and Their Outcomes on Primary-Level Pupils
Source: Int J Environ Res Public Health. 2023 Feb 17;20(4):3575. doi: 10.3390/ijerph20043575 (PMC9965463; doi:10.3390/ijerph20043575)
Supplement: Supplementary file 1 [file ijerph-20-03575-s001.zip › ijerph-2134308-supplementary.pdf]

Table S1. Characteristics of the included studies in the scoping review.

Author (Year)	Country	Sample	Methods	Features of QPE programs	Outcome dimension	Physical measure for ATPE, PAB, MWB and AA	Main findings
Lucertini et al. (2013) [21]	Italy	n=101 (3rd-5th graders). 3 schools assigned. EGs, A (17 M, 21 F) and B (16 M, 21 F), and CG, C (18 M, 8 F).	Six-month, twice-a-week (60 min each session) PE intervention. EGs PE program was age-tailored, included strength training and was administered by specialised teachers. The CG program was not structured and administered by generalist teachers. At baseline and follow-up, a motor and health-related abilities test	(1) Specialist-led pupils benefitted more than generalist led peers.<br>(2) 60-minute PE lessons<br>(3) Structured programme more effective than unstructured<br>(4) Different strength training devices with the same training load more effective.	ATPE and PAB	Measurement of PA: At baseline and follow-up motor and health-related abilities test battery was administered. The tests included- counter movement jump, plate tapping and sit-and-reach, pinch strength and sit-up**.**	Specialist-led pupils demonstrated greater increases in some motor and health-related abilities tests compared to generalist-led peers, while different strength training devices produced comparable increases of strength in both EGs.

			battery was administered.				
Hills et al. (2014) [3]	Australi a	School children in general. There is no specific sample as this is an overview of key issues and challenges for PE and PA promotion in the school system.	N/A	(1) QPE<br>(2) PA during the school day<br>(3) PA before and after school<br>(4) Staff involvement<br>(5) Family and community<br>(6) Comprehensive school program should include: PE and other PA opportunities such as recess, intramurals, interscholastic sports, classroom PA breaks and walk and bicycle to school initiatives.<br>(7) Governments can provide leadership by requiring schools to provide daily PE and other PA opportunities before, during, and after school and by enabling schools to establish health-promoting	ATPE and PAB	None, as this was an overview of research already carried out on PE and PA in schools.	The school setting must embrace their role in public health by adopting a comprehensive school PA program. The crowded school curriculum with an intense focus on AA, lack of school leadership support, funding and resources, plus poor-quality teaching are barriers to PA promotion in schools. The school setting and physical educators in particular, must embrace their role in public health by adopting a comprehensive school PA program.

				environments that support PA.			
Rivera-Pérez et al. (2020) [30]	Spain	n=40 primary pupils (21 girls, 19 boys) 10-12 years (Mage=10.87; SD=0.85), enrolled in two different classes in one school.	The study followed a one group, pre-test-post-test, pre-experimental design. Both classes experienced the same cooperative learning intervention programme conducted in PE, which included two consecutive learning units for a total of 16 sessions (2 per week/50 mins. each). The same PE teacher, an expert in cooperative learning, conducted all sessions.	(1) Cooperative learning as a pedagogical model in PE can contribute to the students' social and emotional learning building quality relationships, learning to manage stressors, and evolving as individuals and as a group.	ATPE, MWB and AA	Cooperative Learning Questionnaire Achievement Goals Questionnaire in PE Emotional Intelligence Questionnaire in PE.	The cooperative learning framework helped increase students' self-approach goals and their emotional control and regulation, and empathy. The present study reinforced the use of cooperative learning in PE, because it can guide students to more adaptive motivational patterns and to develop their emotional intelligence. Furthermore, it contributes to the students' social and emotional learning building quality relationships, learning to manage stressors, and evolve individually and in groups.

Christodoulos et al. (2006) [27]	Greece	The school-based intervention programme was applied to 29 children (18 boys, 11 girls) in the 6th grade of the 2nd Primary School of Agios Stefanos. 49 pupils (24 boys, 25 girls) from the 1st Primary School constituted the CG. The ages were 10-12.5 years.	The intervention programme lasted one academic year and was teacher delivered. The PE instructor conducted both the PA and health components of the programme. The CG did not have any health education intervention. The PE teacher was asked to continue with the formal PE programme during the study. Both groups were measured at the beginning (October) and end (June) of	(1) Seminars to familiarise the teachers with the objectives of the programme. (2) Preparatory teaching material on the basis of successful health promotion programmes was provided.<br>(3) Cooperative activities chosen were enjoyable and fitness oriented. Goal oriented activities were used.<br>(4) PE lessons were complemented with classroom lectures once a week.<br>(5) Computer aided lessons to promote pupils' interaction and entertainment.<br>(6) Parental involvement was encouraged through homework assignments with family activities, by sending educational material home,	ATPE and PAB	(1) Attitudes<br>The planned behaviour theory questionnaire. Pupils indicated their attitudes towards PA and sports participation over the upcoming 12 months on four scales<br>(2) PA<br>The PA recall questionnaire (Aaron et al., 2021) was used. Children were asked to recall all M to V PA, such as organised sports and other leisure time activities, in which they participated at least 10 times during the	Pupils who took part in the intervention had more positive attitudes towards physical activity than the CG and scored significantly more highly on their intention to participate in physical activity. Pupils in the intervention group reported more hours/week spent in organised physical activities than pupils in the CG and a higher proportion of pupils in the intervention classes matched the recommendations of 60 mins. of moderate to vigorous PA daily. Therefore, school health education programmes have the potential to slow the age-related decline in PA and help pupils establish lifelong, healthy PA patterns.

			the academic year.	providing physical activity and nutritional guidelines, and by asking parents to send healthy snacks to school.<br>(7) Promote extracurricular PA, information about community-based sports programmes was disseminated.		preceding year.<br>(3) Intentions<br>Intent was assessed by responses to the following statements: 'I intend to participate in PA three times a week outside of gym class during the next 12 months' and 'I plan to … during the next 12 months'.<br>(4) Anthropometry<br>Age was recorded, standing height and weight was measured.	
Morgan et al. (2008) [20]	Australia	A purposive sampling strategy was used to select 31 teachers for	Mixed-mode design involving data source	(1) Confidence teaching PE	ATPE	Teachers were selected for interviews based on a	This study confirmed that classroom teachers in NSW still experience difficulties teaching PE.

		interviews. 78% female teachers and 21% male teachers. Median age 46–50 years. Average number of years spent teaching was18.4(SD=10.4). Teachers were from both urban (51.8%) and rural (48.2%) settings.	triangulation using semi structured interviews with classroom teachers (n=31) and teacher-completed questionnaires (n=189) from a random sample of 38 schools.	(2) Interest/enthusiasm for PE<br>(3) PE content knowledge<br>(4) Amount of time<br>(5) Equipment availability<br>(6) Quality of facilities<br>(7) Level of Departmental assistance/professional development<br>(8) School executive attitudes towards PE<br>(9) Funds available<br>(10) Class size<br>(11) Attitudes towards PE<br>(12) Perceptions of value of PE<br>(13) Expertise/ qualifications<br>(14) Personal school experiences in PE		range of questionnaire responses describing both positive and negative PE programming practices. Interviews lasted approximately 30–40 mins. A 5-item scale was used to determine teachers' feelings toward PE, which used a 6-point Likert-type scale from 1=strongly disagree to 6=strongly agree.<br>All items were subject to factor and reliability analysis.	A number of major barriers inhibit their efforts and capacity to implement regular and developmentally appropriate PE lessons. This study found the greatest perceived inhibitors were related to institutional factors, such as lack of time/professional development/equipment, which teachers believed were mostly beyond their control. The major adverse effects of these barriers were evident in reduced time spent teaching PE and delivering PE lessons of questionable quality.

Lynch et al. (2017) [22]	Australia	The 73 principal participants were randomly selected to represent a cross-section of Government Primary School communities in a region, where PE responsibility lies with classroom teachers.	Data was gathered using ex-post facto surveys embedded within an interpretivist paradigm.	(1) Teacher competency<br>(2) Children enjoyment<br>(3) Fundamental Movement Skills (4) Human resources<br>(5) HPE equipment<br>(6) Teacher education, supply and development<br>(7) Facilities, equipment and resources<br>(8) Curriculum flexibility<br>(9) Community partnerships<br>(10) Monitoring and quality assurance	ATPE	Questionnaire formulated open ended questions providing principals with the opportunity to express themselves and closed-ended questions where they chose the category that best described their school.	Findings suggest the largest barrier for QPE in primary schools is the qualifications and preparation of teachers. It is recommended that opportunities for developmentally appropriate primary education PE specialisms be provided within degrees, allowing every primary school over time to have a sustain able infrastructure of PE expertise and advocacy.
Tyler G et al. (2016) [23]	United Kingdom	Pupils in K–12 schools	N/A	(1) Teachers provide quality PE<br>(2) Teachers serve as the school PA leader or champion by planning and administering multiple opportunities for PA throughout the school day, before, during and after	ATPE and PAB	N/A as this is a viewpoint article	PE is a process that happens in many places and in the company of many different people. When we adopt this definition of PE, it supports the idea that providing a large quantity of PA experiences before, during and after school is a legitimate way to

				school. (3) Opportunities for PA are needed to help students achieve 60 mins. or more of moderate-intensity PA each day<br>(4) Structured curriculum to help acquire skills, knowledge and dispositions necessary to be "wise consumers" of PA.<br>(5) Students accumulate M, MV, or V PA while on school premises			help more students develop skill and ultimately become physically educated.
Fonyi et al. (2021) [26]	Malaysia	Three types of national schools were selected and the ratio of 3:1:1 stratified random sampling was carried out to determine the sample size of 250 schools out of a total of 372 schools from five randomly selected districts in Selangor.	A mixed-mode survey design was utilised, and the strategy of multiple contact modes and response data collected by a single response mode was applied. The survey had a mixture of open-ended	(1) Academic and Professional Qualification of the principal- when the principals believe in personal development, they will also support the welfare teachers to ensure quality PE learning and teaching experience.<br>(2) Types of schools and years of teaching experience-	ATPE (Leaders)	The Likert scale was used to measure leadership attitude and implementation practices towards PE.	Findings suggest that principals play their roles to create positive PE environments. It is advocated that future research considers a larger geographic area and socioeconomic status of other school districts in other parts of Malaysia to explain the differences found between the different ethnicities, academic and professional

			questions and scale questions.	promoting a positive and quality school learning climate is directly influenced by what a principal has gone through their lifetime as a teacher.<br>(3) PA Level-school principals who are involved in organised physical activity are more likely to possess a higher favourable attitude towards administering quality PE programmes as compared to less active principals.<br>(4) Gender-both female and male principals favour PE and believe in the importance of it.			qualification at Malaysian primary schools. Future research should focus on the investigation of leadership in PE and the intention to further promote PE.<br>ANOVA revealed that the attitude in carrying out PE implementation was significantly different among personal variable groups. The school leaders' attitude towards PE implementation may be dependent on the investigated variables which influence the development and sustainability of quality PE programme.
Wee (2019) [24]	Malaysia	The sample consisted of 1276 teachers from 248 randomly sampled primary schools in Peninsular Malaysia. Majority of PE teachers were young (76.8% below 40 years in age,	This study is survey research designed to obtain research evidence concerning the implementation of PE	(1) Need to attend PE courses.<br>(2) Positioning of PE in the curriculum.<br>(3) Adequate funding from school administrators.	ATPE	Four implementation dimensions were used:<br>(1) Teaching Ability.<br>(2) Administration	There was a need to have specialist PE teachers as majority of the current teachers taught less than five PE periods per week and only 6.2% were PE majors. Independent sample t-tests

		n=980). Almost 94% (n=1197) of them were non-PE majors and 6% (n=79) was PE majors. Almost 71% (n=904) of the PE teachers taught 16 periods per week for other subjects. Majority of them had never attended PE courses (89%, n=1136) and sport related courses (92%, n=1174) since becoming a teacher.	programme from all government-aided primary schools in Peninsular Malaysia	(4) Observations of other teachers PE classes.<br>(5) Administrators discussing PE teaching assignment with teachers.<br>(6) PE classes assigned to teachers based on their professional qualification.<br>(7) Adequate library PE books.		n of PE programme.<br>(3) Class distribution.<br>(4) Non-human factors.<br>Assessed using the Likert Scales.<br>Descriptive statistics were used to report the data from the questionnaire.	conducted on teaching ability revealed that male teachers were more capable than female teachers in knowledge, managing and teaching sport and fitness activities and, detect and correct students' errors.<br>On administration of PE programme, male teachers agreed more than female teachers that administrators organized in-house training, discussed PE teaching assignment, discussed factors affecting PE teaching, and observed PE teaching. As regards to class distribution, male PE teachers agreed more than female teachers that administrators had discussion with them before PE teaching assignment and assignment was based on interest and qualification. T-test

							results also revealed that there were no differences on perception of non-human factor statements
Mohamed et al. (2017) [25]	Malaysia	111 primary school teachers on the PE committee in Selangor were selected through random sampling to answer a questionnaire on Standard Kualiti Pendidikan Malaysia 2010 (SKPM 2010) by the Inspectorate Unit of the Ministry of Education that focuses on the subject of PE (r = .96).	The questionnaire assesses five aspects of education quality: Standard 1: Leadership and Vision, 2) Standard 2: Organisational Management, 3) Standard 3: Curriculum Management, Co-curricular and Sports and Welfare, 4) Standard 4: Learning and Teaching and 5) Standard 5: Student Producibility.	(1) Leadership and vision- status of PE as a subject.<br>(2) Organisational management that covers the needs of the staff, readiness factors such as equipment and sports facilities and financial support.<br>(3) Curriculum management- planning and implementation.<br>(4) Process of teaching and learning- high skilled teaching, well planned learning and teaching processes.<br>(5) Student producibility- high student engagement.	PE Implementation in schools	Questionnaires	Each of the five aspects (see 'Quality Features of PE Prog.' list) influence the level of PE implementation. This affects the level of achievement of the PE objectives as a whole, particularly in developing motor skills at an early-stage fundamental to a healthy lifestyle. Failure to support the PE program will weaken student interest in PA, because poor physical fitness, lead to poor self-confidence, and an increase of health problems.<br>The samples obtained from the 111 respondents, 94 showed a medium level of implementation (83.9%) while only 7 (6.3%)

							schools performed at a low level, and 10 (8.9%) at a high level.
Kulik (2009) [17]	USA	39 of the 91 public high schools in Allegheny, Beaver, Indiana and Westmoreland counties were recruited to participate in this cross-sectional quantitative study.	A quantitative, cross-sectional study design was used, and a survey instrument was developed specifically for the use in this study.	(1) Providing students with learning experiences that meet individual developmental needs, which help to improve mental alertness, academic performance, readiness to learn and enthusiasm for learning (NASPE).<br>(2) Instruction time- 45 mins. per day x 5 days per week = 225 mins. per week.<br>(3) Teacher qualification and professional development- PE is delivered by certified/licensed physical education teachers in an appropriate play space.<br>(4) Physical education specialists teach longer lessons, spend	QPE programme	The online survey assessed time requirements for PE, teacher qualifications, and instructional facilities that the (NASPE) has identified to be important for offering QPE programs.	The results suggest that high schools in Southwestern Pennsylvania do not offer PE daily and do not offer the recommended 225 mins. of PE per week. It does not appear that the factors recommended by NASPE influence the amount of time that is allocated for PE instruction. However, this does not suggest that these factors do not influence PA time within PE. Thus, it may be necessary to revise the NASPE guidelines for QPE to include participation in PA rather than duration of instruction time.

				more time on developing skills, impart more knowledge and provide more MVP than do classroom teachers.<br>(5) Availability of PE facilities- facilities such as open fields, outdoor obstacle course, playground equipment and room with cardio and weightlifting equipment were associated with increased participation in PA.			
Roux (2020) [18]	South Africa	Cohort of principals, Head of Departments and Learning Support teachers responsible for PE in selected South African public primary schools. These participants were drawn from selected quintile 1–3 schools in rural and township areas, and quintile 4–5 schools in urban areas.	The mixed-methods approach (quantitative: questionnaires and qualitative: semi structured interviews and focus group discussions) was used to	(1) Quality physical resources through building new facilities and upgrading existing ones.<br>(2) Train educators on PE teaching and the use and maintenance of facilities.<br>(3) Teacher to be knowledgeable about the school curriculum, unpack	PE Implementation and quality programmes	Structured interviews and focus group discussions.	The findings revealed that the educators from quintile 4 and 5 schools especially are of the opinion that challenges, such as a lack of resources, qualified PE specialist educators, and facilities and equipment negatively affected the delivery of PE at their schools. Participants perceived that there are varying

			collect data. The purposive sampling method was used to select the participants.	its specialised content, use available resources appropriately and design suitable learning programmes.			contextual and socio-economic school settings affecting the delivery of PE in the Curriculum Assessment Policy Statement (CAPS) curriculum. The researcher recommends that all learners have access to the adequate provision of PE programmes.
Murphy et al. (2012) [28]	Ireland	Questionnaire survey of all tutors (n=26), interviews with a convenience sample of tutors (n=6), observation of all tutors as they engaged with the tutor professional development programme (TPDP) and observation of the sample as they taught PE in their own schools and facilitated the National In-service PE Programme (NIPEP)	A pragmatist theoretical framework allied to the context input process product evaluation model using a mixed methods research design were chosen to underpin the study.	(1) Quality preparation of tutors acknowledging the advantage of technology that may provide a new and cost-effective way of supporting them. (2) Retaining the practical exploration of content by both tutors and teachers that prompts reflection on the nature and content of programmes of PE. (3) Teachers' attitudes. (4) Changes in their understanding of PE	ATPE	A questionnaire survey, interviews and observations were used.	The PE tutors were satisfied that the (TPDP) prepared them for facilitation of the (NIPEP), they felt both competent and confident as they embarked on facilitation of the initiative. The teachers who evaluated the first phase of the NIPEP were satisfied that it had provided them with renewed impetus to implement the PE Curriculum.

				and the PE Curriculum.<br>(5) Willingness to teach new elements of PE content.<br>(6) Attitudes to meeting the individual needs of children in PE.			
Coulter et al. (2012) [19]	Ireland	A single school case study was employed (n=28) teachers, (n=780 pupils) and all teachers and a sample of children participated in focus group interviews.	Qualitative data were collected through two focus group semi-structured interviews with groups of teachers and groups of pupils. Testing took place before and after the PE-PDP. A digitally recorded, daily journal was kept throughout the process.	(1) Resources and material - All teachers reported that the resources provided were invaluable in supporting and enabling their teaching.<br>(2) Modelling - Teachers commented that having the opportunity to actually see another teacher in action, modelling the content and strategies for them was extremely helpful.	ATPE	The process of qualitative data analysis began with the transcription and reading of the data generated. All transcripts were categorised and themes using systematic content analysis were generated. In Basic Moves PE CPD courses, administrators provoked critical and even	Post the PE-Professional development programme, teachers' PE content knowledge had expanded, and this encouraged them to use existing classroom pedagogical strategies in the PE context. This developed their confidence in teaching PE and a greater understanding of the PE curriculum and its purpose.

						uncomfortable discussions in order to challenge teachers' pre-existing views of PE. The group organised and emerged as a 'community of practice'.	
Atencio et al. (2021) [29]	Scotland	The study followed 10 teaching professionals, specialists and local authority managers working with the DPEG's1 Basic Moves programme for a period of 10 years. All participants were recruited because they had attended several Basic Moves National CPD Courses.		(1) Collaborative learning.<br>(2) Communities of practice.<br>(3) Teacher learning/ Professional development.<br>(4) Complex educational systems.	ATPE	In Basic Moves PE CPD courses, administrators provoked critical and even uncomfortable discussions in order to challenge teachers' pre-existing views of PE. The group organised and emerged as a 'community of practice'.	Findings suggest that the PE practitioners in this study worked alongside a range of educational stakeholders within a broader 'nested system' that was constantly evolving and changing. Contemporary PE CPD must challenge practitioners to become critical and innovative learners in the context of dynamic learning communities. This version of PE CPD requires sustained support at the local level and directly involves PE

							practitioners, their peers, and local authority leadership in the planning and operational phases.
